# Supplementary material for: Interventional embolization combined with surgical resection for treatment of extracranial AVM of the head and neck: A monocentric retrospective analysis
Source: PLoS One. 2022 Sep 1;17(9):e0273018. doi: 10.1371/journal.pone.0273018 (PMC9436082; doi:10.1371/journal.pone.0273018)
Supplement: S1 Supplement — (PDF) [file pone.0273018.s001.pdf]

Aachen Questionnaire for the treatment of Extracranial vascular Malformations

Name, First Name	
Date of Birth	
Date of Examination	

PRIMARY EXAMINATION – to be filled in by physician

1-E **Indicate the location and full extension of the lesion on the illustration.**

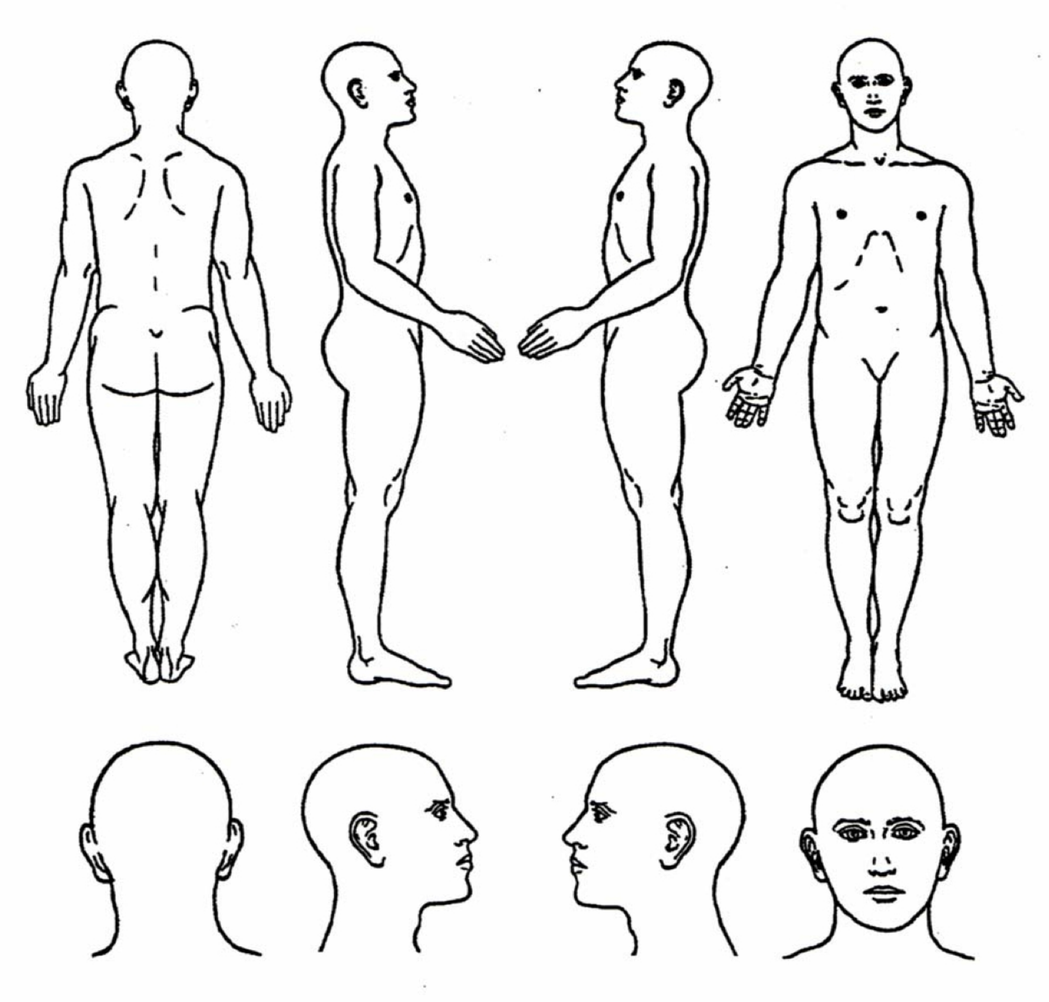

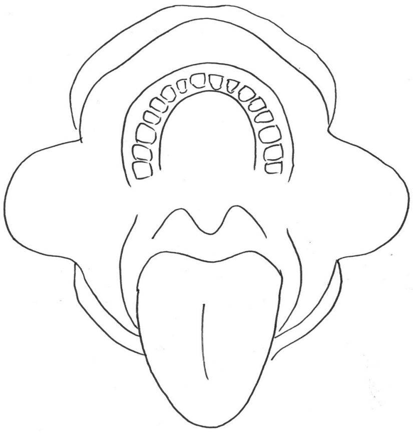

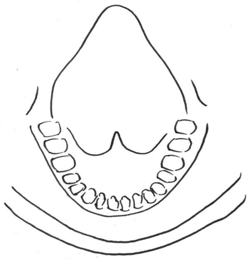

2- E **How old was the patient when the lesion was first noticed?**

3- E **Please describe the changes and the course of progression from the lesion.**

4-E **Did the volume of the lesion change over the last 10 years in proportion to the rest of the body?**

◯ Yes ◯ No

5-E **Have there been episodes of bleeding from the lesion?**

◯ Yes ◯ No

6-E **Did the bleedings have to be treated by medical staff?**

◯ Yes ◯ No

7-E **If yes, how were the bleedings treated? Please describe.**

8-E **Has there been any discharge of clear fluid from the lesion?**

◯ Yes ◯ No

9-E **Does the colour of the skin around the lesion change e.g. under physical strain or when below the heart?**

○ Yes ○ No

10-E **Does the volume of the lesion increase e.g. under physical strain or when below the heart?**

○ Yes ○ No

11-E **Are there palpable masses or concrements in the lesion?**

○ Yes ○ No

12-E **Is there noticeable thrill or pulsation in the lesion?**

○ Yes ○ No

13-E **Is there paraesthesia or alteration of sensation in or around the lesion?**

○ Yes ○ No

14-E **Is there any pain at the lesion?**

○ Yes ○ No

15-E **Has the patient been under treatment due to the lesion?**

○ No

○ Yes, at another medical institution. Please state the name of the institution:

16-E **How many treatment sessions has the patient had?**

○ 1-2 ○ 3-5 ○ 6-1 ○ >10

17-E **Please state what treatment procedure (e.g. surgery, embolization, laser) was applied at what time.**

	Modality	Date (Month/Year)
**Procedure 1**		
**Procedure 2**		
**Procedure 3**		
**Procedure 4**		
**Procedure 5**		
**Procedure 6**		
**Procedure 7**		
**Procedure 8**		
**Procedure 9**		
**Procedure 10**		
**Further**		

18-E **Where would the patient like to see improvements after the lesion has been treated? Check all that apply.**

- ○ Pain
- ○ Swallowing
- ○ Chewing
- ○ Speech
- ○ Aesthetic appeal
- ○ Vision
- ○ Hearing
- ○ Paraesthesia
- ○ Movement
- ○ Bleeding
- ○ Mood / Well-being
- ○ Other
- ○ Other
- ○ Other

19- E **Does the patient have any other medical conditions (Chronic illness, Coagulopathy, Vascular disease etc.) or hereditary conditions (Genetic alterations, Syndromes etc.). If so, please specify:**

20-E **What medication does the patient take? Please specify dosage and frequency.**

21-E **Does the patient have any allergies? Please list and describe the reaction to those agents.**

22-E **Is there anything else the patient would like us to know about the lesion or past treatment?**

23-E **How did you come to our institution?**

○ Referral from family physician

○ Name/Speciality of referring physician

○ Other (e.g. recommendation)

24-E **Clinical Diagnosis**

○ Venous Malformation (VM)

○ Arteriovenous Malformation (AVM)

○ Infantile Capillary Haemangioma

○ Lymphatic Malformation (LM)

○ Mixed Malformation (VM/LM)

○ Other vascular malformation. Specify:

○ Other diagnosis. Specify:

Aachen Questionnaire for the treatment of Extracranial vascular Malformations

Name, First Name	
Date of Birth	
Date of Examination	

FOLLOW-UP EXAMINATION – to be filled by the patient

1-W **Where would you like to see improvements after the lesion has been treated? Check all that apply.**

- ○ Pain
- ○ Swallowing
- ○ Chewing
- ○ Speech
- ○ Aesthetic appeal
- ○ Vision
- ○ Hearing
- ○ Paraesthesia
- ○ Movement
- ○ Bleeding
- ○ Mood / Well-being
- ○ Other
- ○ Other
- ○ Other

2-W **Where have you seen improvement with your current treatment? Check all that apply.**

- ○ None
- ○ Pain
- ○ Swallowing
- ○ Chewing
- ○ Speech
- ○ Aesthetic appeal
- ○ Vision
- ○ Hearing
- ○ Paraesthesia
- ○ Movement
- ○ Bleeding
- ○ Mood / Well-being
- ○ Other
- ○ Other
- ○ Other

3-W **How satisfied are you with the treatment so far?**

○ Very satisfied ○ Mostly satisfied ○ Undecided ○ Mostly dissatisfied ○ Very dissatisfied

4-W **Please describe any adverse effect or complications caused by the treatment:**

5-W **Are you going to continue the treatment at our institution?**

○ Yes

○ No, the treatment was successful.

○ No, I will continue treatment at another institution.

○ No, I am not satisfied with the treatment.

○ No, other reasons. Please specify:

Thank you very much for taking the time to answer these questions.

If we missed anything in the questionnaire, or if there is anything else you'd like to tell us, please write your comments in the box below.
