# Supplementary material for: Interventional embolization combined with surgical resection for treatment of extracranial AVM of the head and neck: A monocentric retrospective analysis
Source: PLoS One. 2022 Sep 1;17(9):e0273018. doi: 10.1371/journal.pone.0273018 (PMC9436082; doi:10.1371/journal.pone.0273018)
Supplement: S3 Supplement — (DOCX) [file pone.0273018.s003.docx]

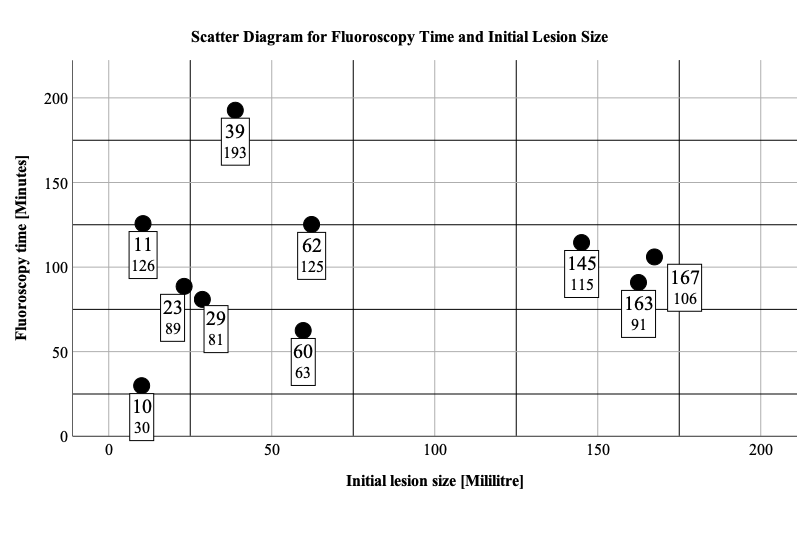


**Supplement 3. Scatter diagram for correlation between fluoroscopy time and initial lesion size (p=0.814).**
