# Supplementary material for: Interventional embolization combined with surgical resection for treatment of extracranial AVM of the head and neck: A monocentric retrospective analysis
Source: PLoS One. 2022 Sep 1;17(9):e0273018. doi: 10.1371/journal.pone.0273018 (PMC9436082; doi:10.1371/journal.pone.0273018)
Supplement: Attachment — Submitted filename: Response to Reviewers.docx [file pone.0273018.s006.docx]

Dear Editors,

Dear reviewers,

We would like to thank you for the thoughtful and favorable reviews and the opportunity to revise our manuscript. We believe that our manuscript has benefitted considerably from the remarks, and we hope that we addressed the mentioned issues to your satisfaction.

Below you can find our responses to the reviewer’s comments in red. Please also find attached our revised manuscript in two versions: with and without tracked changes.

Additionally, we have added the full data set of our study as supplement S5 file.

We are happy to hear from you if you have any other question.

With our highest regards,

The authors

Reviewer #1: […]
Title: As this is a retrospective analysis, how can it be an intention to treat analysis? In my opinion this is only possible in prospective studies.

We would like to thank the reviewers as he/she accurately points out that an intention-to-treat analysis is defined as a prospective study design. We have made according changes in the title:

Interventional embolization combined with surgical resection for treatment of extracranial AVM of the head and neck: A monocentric retrospective analysis

Abstract:
“Patients treated according to our protocol show a high satisfaction rate, regardless of the radiographic outcome.” Please use past tense (showed)

We would like to give thanks to the reviewer for the correction of this mistake. We changed the sentence into past tense.

Patients treated according to our protocol showed a high satisfaction rate, regardless of the radiographic outcome.

(Clean copy lines 52-53)

Advances in knowledge:
In my opinion the statement is too strong, please consider changing it to “..might be an effective..”

In consideration of the small sample size and the rarity of the condition, the reviewer is correct that a less strong wording might be helpful to stimulate a discussion.

The results of this study indicate that embolization followed by surgical resection might be an effective treatment for extracranial AVM of the head and neck and is accompanied by high patient satisfaction.

(Clean copy lines 57-59)

[…]
Sometimes the mean is used and sometimes the median. It should be checked if in cases of mean normality is given.

We would like to thank the reviewer for the helpful remark to check the usage of the mean and median. We used the mean for statistical analysis wherever the distribution of data was expected to be symmetrical (e.g. patients’ age, lesion volume, accumulated fluoroscopy time), or if reference scores where provided as such (e.g. in the UW-QOL v4 questionnaire). The median was used only in reference to the hospitalization time, where outliers could be identified as shown in the comprehensive overview of the study group (Table 2). In careful attention of these statistical aspects, we deem the utilization to be appropriate.

Reviewer #2:  […]

Two statements seem unclear and could be enhanced with minor revisions:

- In line 169, you state that surgery was performed within seven days of the embolization procedure; however, in line 238, the range for the time between procedure and surgery is 1-14d.

The reviewer is correct to point out the mismatching specification of time between embolization procedure and surgery. The passage on the surgical procedure merely tries to give an overview on the course of action of the procedure. The timeframe of seven days was communicated between the neuroradiological and surgical department to facilitate smooth workflow. To prevent the number in this section to be confused as part of the results, we erased the section.

Surgical intervention was performed by the Department of Oral and Maxillofacial Surgery at University Hospital RWTH Aachen.

(Clean copy lines 165-166)

- in your results (line 242), you state that five of eight patients were considered cured, but in supplement four, there seem to be four in the whole group and four in the incomplete group (Misplacement of Case 9?); Does that influence the statistical calculations as well?

We would like to thank the reviewer for this very important note. There was indeed a mistake in the scatter diagram and case #9 was wrongfully placed in the “incomplete” group. This has no effect on the calculations as they were performed independently. The revised copy of the scatter diagrams can be found in file S3 Supplement 3 and file S4 Supplement 4.

**Supplement 3. Scatter diagram for correlation between fluoroscopy time and initial lesion size (p=0.814).**

**Supplement 4. Scatter diagram for correlation between fluoroscopy time and radiographic outcome (p=0.895).**

Reviewer #3:

- With your Abstract section, please please provide as much information as possible within the allowed 300-word limit. Add p values with your most prominent results. Style must be "(p < 0.001)", or "(p = 0.012)".

We are happy for the reviewer to bring this aspect to our attention. As most of the findings are of descriptive nature and significance of comparison had lower priority in the analysis, the highlights of the results are listed in the abstract. We have added the significant decrease of symptoms (p=0.002) as another prominent finding.

Radiographic resolution was achieved in 62.5% of cases and a significant decrease of symptoms was identified (p=0.002). None of the patients reported dissatisfaction and no major complications occurred.

(Clean copy lines 45-47)

- Reference style has to be adapted to the Journal guidelines. "(...) and can be evident at birth.(1)" must read "(...) and can be evident at birth¹."

We would like to thank the reviewer for this remark. All citations have been adapted accordingly.

- With ALL materials (including chemicals) and methodologies (including statistical software), please use general names with your text, followed by (brand name; manufacturer, city, St[ate, abbreviated, if US], country) in parentheses. Stick to semicolon. Revise thoroughly.

The reviewer brings up a very good point that interrupted the flow of reading significantly. We have corrected the sections according to the recommendations.

- With your Conclusions, please stick exclusively to your revised aims. Do not simply repeat your results here. Do not speculate on future studies. Instead, provide a reasonable and generalizable extension of your outcome.

This remark by the reviewer was very helpful for the authors to reconsider the key findings of the article and sparked a fruitful discussion. With the presented treatment protocol and its results, we are certain to only show one possible way of managing this rare condition. In view of existing literature, the conclusion of the article may best be as cautious as outlined in the current manuscript to give room and motivation for further studies. However, if changes in the regard to this aspect are needed, we are happy to adapt the manuscript.

- Please revise your reference list for uniform formatting. Style would be "Kasraei S, Sami L, Hendi S, Alikhani MY, Rezaei-Soufi L, Khamverdi Z. Antibacterial properties of composite resins incorporating silver and zinc oxide nanoparticles on Streptococcus mutans and Lactobacillus. Restor Dent Endod. 2014; 39(2): 109–114. <https://doi.org/10.5395/rde.2014.39.2.109> PMID: 24790923" Provide doi and PMID numbers.

The reviewer is correct to bring this point to our attention. We have made changes in the bibliography according to the recommendations in the PLOS submission guidelines. We provided the PMID if DOI could not be obtained.
